# Time of recovery and associated factors of children with severe acute malnutrition treated at outpatient therapeutic feeding program in Dire Dawa, Eastern Ethiopia

**DOI:** 10.1371/journal.pone.0217344

**Published:** 2019-06-13

**Authors:** Binyam Atnafe, Kedir Teji Roba, Tariku Dingeta

**Affiliations:** 1 Dire Dawa City Administration, Dire Dawa Regional Health Bureau, Dire Dawa, Ethiopia; 2 School of Nursing and Midwifery, College of Health and Medical Sciences, Haramaya University, Harar, Ethiopia; 3 Department of Public Health, College of Health and Medical Sciences, Haramaya University, Harar, Ethiopia; ESIC Medical College & PGIMSR, INDIA

## Abstract

**Background:**

The outpatient therapeutic feeding program is one dimension of the Community Based Management of Acute Malnutrition (CMAM) that provides screening, diagnostic and treatment services for children with Severe Acute Malnutrition (SAM). However, little is known about the program outcomes and factors affecting time to recovery.

**Objectives:**

To determine median time of recovery and associated factors among under-five children with SAM treated at outpatient therapeutic feeding unit in Dire Dawa, Eastern Ethiopia from January 1^st^, 2013 to December 31^st^, 2016.

**Methods:**

A facility-based retrospective cohort study supplemented with qualitative inquiry was conducted to analyze the records of 713 under-5 children with SAM that were randomly selected from four health centers and one hospital in Dire Dawa. In-depth interviews were conducted with five health professionals. Data was collected from the nutrition registration log book by using structured check lists. The collected data were entered into EPI-data version 3.1 software and exported to SPSS version 23 for analysis using Kaplan Meir and Cox proportional hazard regression.

**Results:**

The overall recovery rate was 569 (79.8%). Eighty (11.2%) defaulted, 27 (3.8%) were non-responders, 4 (0.6%) died and 15 (2.1%) were transferred-out. The median recovery time was 8.7 weeks (IQR: 5.0–14 weeks). Children with an admission weight of ≥7kg (AHR = 1.73, 95% CI: (1.41–2.14), children who were dewormed (AHR = 1.44, 95% CI: (1.01–2.06) and children with weight gain of ≥8g/kg/day (AHR = 5.76, 95% CI: (4.51–7.38) had higher probability of recovering faster. However, marasmic children stayed longer in treatment (AHR = 0.51, 95% CI: (0.37–0.71) and a low Plumpy Nut consumption rate (g/day) (AHR = 0.79) was associated with longer time of stay on treatment.

**Conclusion:**

The recovery rate was within the level specified in the Sphere International standards which is >75%. A higher weight at admission, taking deworming and a steady weight gain were positively associated with a fast recovery time. Appropriate nutritional therapy and management of SAM as per the national protocol will be helpful to overcome lower weight gain and higher length of stay on treatment.

## Introduction

SAM is the most noticeable type of undernutrition. Children with SAM have severe wasting, weight-for-height z-scores of < –3 [1] or MUAC < 110mm. They may also present with nutritional edema characterized by a swollen face, feet and limbs. Edema is a life-threatening condition requiring urgent treatment [1,2].

SAM is responsible for 21% of disability-adjusted life-years (DALYs) for children younger than 5 years [3]. SAM not only has direct nutritional consequences but also causes long-term effects such as lower intelligence quotient (IQ) and stunted growth [1,4]. Malnutrition also drastically reduces the functioning of the immune system, increasing children’s vulnerability to other diseases. The risk of death increases among malnourished children with diarrhea, pneumonia, measles and malaria morbidity [3]. As a report in Ethiopia showed, 24.3% children with SAM developed pneumonia while many contracted diarrhea (21%) and tuberculosis(11%) [5]. Deficiencies of vitamin A and zinc were also estimated to be responsible for 0·6 million and 0·4 million deaths, respectively [3].

Some 85% of severely wasted children with no medical complications can be treated at home in outpatient care without the need for inpatient care at a health facility [6]. This has the advantage of protecting them from exposure to infections and allows mothers to attend to the rest of the family while providing care for their malnourished child [6]. Outpatient therapeutic feeding programs (OTPs) are part of the routine health care services in Ethiopia, provided at health facilities. The program services include diagnoses of children with SAM with in health facilities and at community level by Community Health Workers (CHWs), provide ready-to-use therapeutic foods (RUTF) which runs every week to eat at home and a course of routine medications including amoxicillin, folic acid, vitamin-A, measles vaccine and deworming [7].

Malnutrition is still a public health concern in Ethiopia and as the Ethiopian Demographic and Health Survey reported in 2016 shows 38% of children under the age of five were stunted, 24% were underweight and 10% were wasted. Currently, severe wasting among under-five children in Dire Dawa region is 4.2%. This figure is higher in severe wasting compared to the national figure which is 3% [8].

Regardless of the presence of OTP and other nutrition programs in the country, the national survey (EDHS) and different studies have showed that nutritional problems still remain a major concern in Ethiopia [8,9]. There are studies conducted in Ethiopia to assess the recovery time and its predictors among children with SAM [10,11,12,13]. However, the majority of these studies did not assess the effect of appropriate consumption of Plumpy nut per weight of the child and were unable to show clearly the effects of some factors to the recovery time. As the factors vary according to context, this study assessed predictors of recovery time in this particular area which can have positive message for the concerned bodies working in this area. Thus it will add value for possible predictors of recovery time and indicate intervention to improve the program. Therefore, the aim of this study was to fill gaps by assessing the magnitude of recovery rate, the median time of recovery from SAM and factors affecting time to recovery.

## Materials and methods

### Study settings

This study was conducted in Dire Dawa which is located in Eastern Ethiopia. Dire Dawa has a total population of 453,000 with approximately 41,767 being under-five children. 285,000 (68%) lives in urban and 168,000 (32%) lives in the rural areas [14]. There are six hospitals (2 public, 3 private and 1 Ethio- Djibouti), 15 Health centers (8 in town and 7 in rural) and 11 health posts (3 in town and 8 in rural) with 100% health service coverage, in Dire Dawa [14]. All health centers provide outpatient therapeutic feeding service and growth monitoring.

### Study design and population

A facility-based retrospective cohort study supplemented with qualitative inquiry was conducted. The source population was all records of SAM patients, aged 6–59 months who were enrolled in OTP from January 2013 to December 2016. The study period of this research work was from February to March in 2017 as of data collection period. Records of children with SAM aged 6–59 months who were treated under OTP within specified period were randomly selected. Those with incomplete medical records on outcome variables and anthropometric measurements were excluded. Data were collected from four health centers and one hospital that were selected randomly.

### Sample size determination and sampling procedure

**Fig 1:**
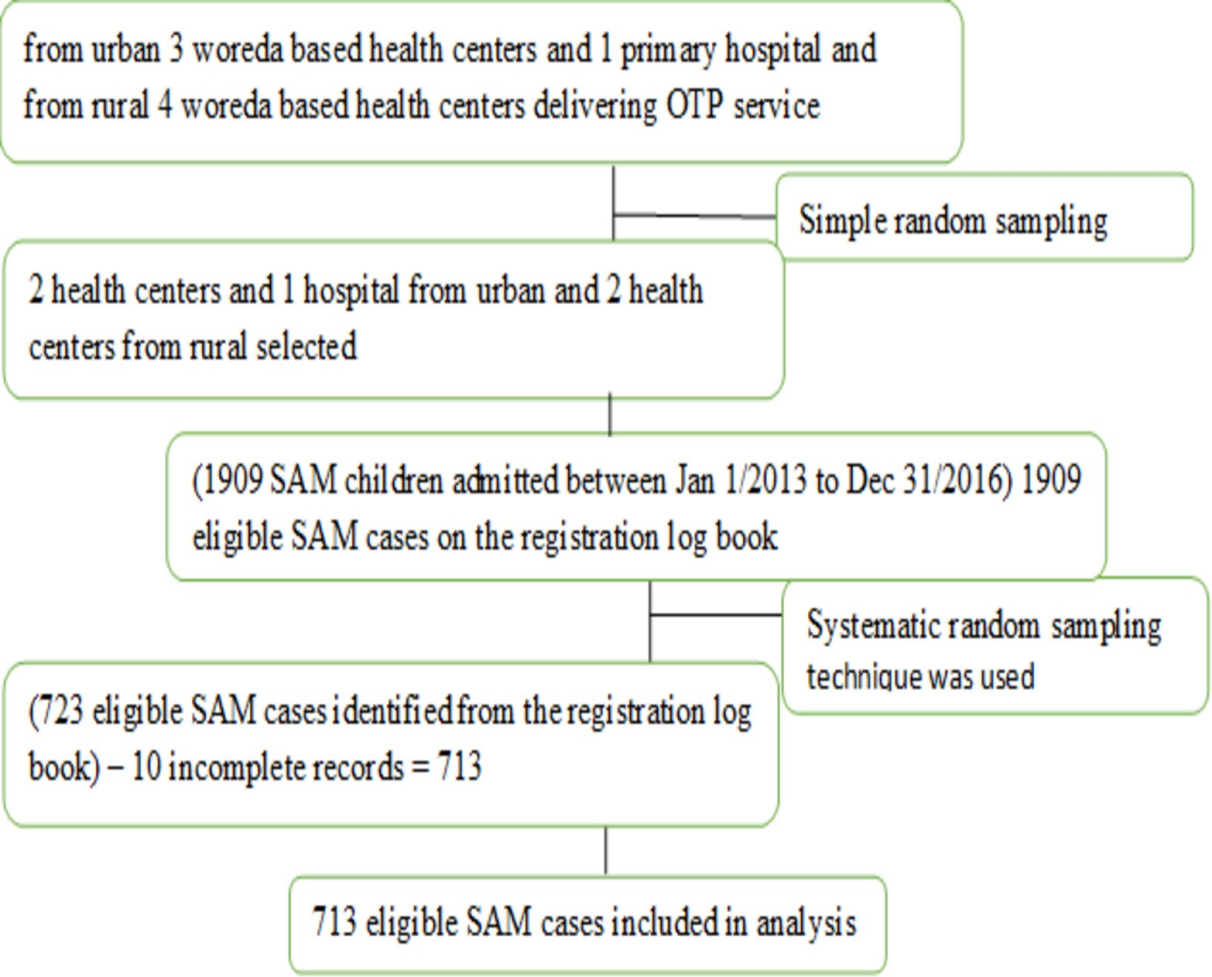
Sampling scheme of severe acutely malnourished children included in the study. Simple random sampling was used to select health centers. In the selected health centers, systematic random sampling was used to select eligible SAM children. SAM refers to Severe Acute Malnutrition and OTP refers to Outpatient Therapeutic Care Program.

To determine the recovery rate of under-five children with SAM, a single population proportion formula was used which provided the maximum sample size of 723. This sample size incorporated a 10% incomplete record rate and design effect of 2.0. To determine factors affecting time to recovery among under-five children with SAM, the sample size was calculated based on the formula for the two population proportions using open Epi version 2.3.1 [15]. The total sample size to detect the factors affecting time to recovery was 668 (including 10% for incomplete records and design effect 2.0). The maximum sample size of 723 was taken as the final sample size. The total sample size was proportionally allocated for all selected health facilities providing OTP in the study area based on the number of children with SAM admitted to OTP services in each facilities. Cases of SAM were then selected from the OTP register book and patient’s cards enrolled from January 2013 to December 2016. A systematic random sampling technique was used to get an individual sampling unit at the health facility level (Fig 1). In-depth interview was conducted with five purposively selected health professionals. First the list of staffs with their working department were obtained from head of the health centers, then based on their working department; three female Bsc in Nursing and one male and one female diplomas in Nursing were included in the interview.

### Data collection method and data quality control

Data were collected from the OTP register book starting from January 2013 to December 2016 by using semi-structured and pre-tested check lists on 5% of the sample size outside the study area. Data was gathered by five female nurses and supervised by two health professionals (BSC Nurses) who were not employed yet.

Training was given by the principal investigator for one day before data collection period about the objectives of the study, variables on the data abstraction sheet, OTP cards and how to retrieve data for this study. To ensure quality data collection, close supervision was carried out by principal investigator and a supervisor during data abstraction. The completed data abstraction form was checked for completeness of information by supervisors and principal investigator on daily bases.

A face to face interview was conducted by one individual with BSc qualification in nursing. Repeated ideas were raised with the fifth interviewee and saturation was reached at this conversation. The interview was stopped after interviewing five Health professionals. The investigator was in close contact with them and was involved when there was any unclear question.

### Variables and measurements

The primary outcome measured in this study is recovery from SAM after initiation of OTP and considered as recovered. Individuals defaulted (patient that is absent for two consecutive weeks and confirmed alive by a home visit), outcome unknown (patient absent for three consecutive weightings in outpatient care), non-response (patient that has not reached the discharge criteria after two months in the outpatient program), died and transfer out to other facility were considered as censored (not-recovered). Finally, the out-come of each subject was dichotomized in to recovered and censored (not-recovered). Other various outcome measures considered were average length of stay, average rate of weight gain, cure rate, death rate, and default rate. Recovery is defined as children that has reached the discharge criteria. i.e. MUAC ≥ 12cm or W/L> = 85% or W/H> = 85% on more than one occasion for children with marasmus and no edema regardless of their body weight status for 14 days for kwashiorkor cases.

**Dependent variable** was time to recovery.

**Independent variables** were age of children, sex of children, residence and referred from (hospital, HEW or self), anthropometry measurements, medical co-morbidities, intake of routine medication, clinical form of SAM, admission status (new, readmission or return after default) and follow-up characteristics such as follow-up anthropometry measurements and clinical features and routine medications.

### Operational definitions

**Recovered:** Patient that has reached the discharge criteria.

**Discharge Criteria:** Age 6 month-5years- W/H> = 85% on more than one occasion or MUAC ≥120mm (Two weeks for out-patients) and no oedema for 14 days (out-patient) for edematous,

**Average weight gain:** sum of weight gains/No of children 6–59 months who were cured.

**Average length of stay:** sum of length of stay/No of children 6–59 months who were cured.

**RUTF consumption appropriate for weight:** appropriate amount of RUTF was calculated based on the national SAM management protocol standing from weight of respective children.

**Weight gain (g/kg/day):** is average weight (in gram) increase for every Kg of body weight of the child per day. It is determined by;

Individual weight gains in marasmic patients were calculated with:

$$\frac{dischargeweight(g)-admissionweight(g)}{admissionweight(kg)\times no.of\;days\;beteween\;minimum\;weight\;and\;discharged\;weight}$$

Data processing and analysis

Data were entered in to EpiData version 3.1 software and analyzed using SPSS version 23. Survival curve and hazard curve was used to display the survival (time to recovery) among different characteristics. Log rank test was done to compare median recovery time among different groups. Cox proportional hazard model with both bivariate and multivariate analysis was done. P-value < 0.05 was considered as statistically significant.

### Ethical consideration

Ethical approval was obtained from Haramaya University, College of Health and Medical Sciences,’ Institutional Health Research Ethics Review Committee (IHRERC). The study was conducted in collaboration with Dire Dawa Administrative Regional Health Bureau. Consents were also obtained from Regional Health Bureau and health facilities. In the existing system of the nation, there is no way to communicate with the patients once they get discharged from the treatment. As such, informed consent from the parents/caregivers of the children was not obtainable. The study didn’t give any supplementary interventions to the participants. As it was conducted based on the OTP records, the consent relied on the ethical board’s approval and written consent from respective Regional Health Bureau and health facilities. Furthermore, the research ethical board was also aware of the issue before its approval that informed consent couldn’t be obtained from the study participants. In order to protect the confidentiality of information, name and other identification were not included in questionnaires and to maximum effort was maintain privacy of the patient cards.

## Result

### Socio-demographic and admission characteristics of children

Of 723 randomly selected out-patient record cards, 10 were discarded due to incompleteness. The mean (±SD) age of children in the program was 19.93 (±10.855) months. 359(50.4%) of the children were females and 403 (56.5%) of the children were enrolled in urban health centers. Ninety percent of children included in the study were wasted (marasmic) while the rest had edema (9.1%). The overall median weight of children at admission was 6.5 kg (Inter quartile range: 5.6 to 7.5 kg) (Table 1).

**Table 1:** Socio- demographic and admission characteristics of children with SAM enrolled to OTP in Dire Dawa, Ethiopia; from January, 2013 to December, 2016.

Socio-demographic and admission characteristics	category	Frequency number (%)
**Child’s age (month)(n = 713)**	< or = 24 months	558(78.3%)
	>24 months	155(21.7%)
**Residence (n = 713)**	Rural	310(43.5%)
	Urban	403(56.5%)
**Sex (n = 713)**	Male	354(49.6%)
	female	359(50.4%)
**Admission weight**	< 7 kg	427 (59.9%)
	≥ 7 kg	286 (40.1%)
**Admission type(n = 713)**	New admission	688(96.5%)
	Re-admission	25(3.5%)
**Clinical form of SAM (n = 713)**	Edema	65(9.1%)
	Wasted (Marasmus)	648(90.9%)
**Breast feeding(n = 559)**	Yes	391(69.9%)
	No	168(30.1%)
**Referred from(n = 713)**	Hospital	3(0.4%)
	OPD	300(42.1%)
	HEW	187(26.2%)
	Self/parents/	223(31.3%)
**Length of stay (n = 713)**	≤ 6 weeks	308(43.2%)
	> 6 weeks	405(56.8%)

### Co-morbidities and routine medications

The most common co-morbidities accompanied with SAM at admission were diarrhea (31.1%), anemia (25.4%), fever (10.2%), vomiting (8.6%), superficial skin infection or skin peeling (0.7%), HIV infection (0.5%) and TB (0.2%). HIV testing was done only for 411 children. No children with malaria and hypothermia were found on the records of children with SAM (Table 2).

**Table 2:** Medical co-morbidity characteristics of children with SAM on admission under OTP in Dire Dawa, Ethiopia; from January, 2013 to December, 2016.

characteristics	category	Frequency number (%)
**Diarrhea (n = 713)**	present	222(31.1%)
	absent	491(68.9%)
**Diarrheal type (n = 222)**	watery-diarrhea	217(97.7%)
	bloody-diarrhea	5(2.3%)
**Diarrhea by duration(n = 222)**	acute	218(98.2%)
	persistent	4(1.8%)
**Cough (n = 713)**	present	220(30.9%)
	absent	493(69.1%)
**Anemia (n = 713)**	present	181(25.4%)
	absent	532(74.6%)
**Fever (n = 713)**	Present	73(10.2%)
	absent	640(89.8%)
**Vomiting (n = 713)**	Present	61(8.6%)
	absent	652(91.4%)
**Skin infection (n = 713)**	Present	5(0.7%)
	absent	708(99.3%)
**HIV infection (n = 411)**	present	2(0.5%)
	absent	409(99.5%)
**TB (n = 504)**	present	1(0.2%)
	absent	303(99.8%)

Out of 713 children whose medication records were available for review, 493 (69.1%) of the children were given amoxicillin while vitamin A (45.6%), measles vaccine (46.2) and folic acid (5.8%) (Table 3).

**Table 3:** Treatments given information to children with SAM enrolled to OTP in Dire Dawa, Ethiopia; from January, 2013 to December, 2016.

Treatment given	category	Frequency number (%)
**Amoxicillin/Antibiotics (n = 713)**	Yes	493(69.1%)
	No	220(30.9%)
**Vitamin A (n = 713)**	Yes	325(45.6%)
	No	388(54.4%)
**Measles (n = 654)**	Yes	302(46.2%)
	No	352(53.8%)
**Albendazole/mebendazole (n = 590)**	Yes	148(25.1%)
	No	442(74.9%)
**Folic acid (n = 713)**	Yes	41(5.8%)
	No	672(94.2%)

After collecting data on the amount of Plumpy Nut sachets consumed by the children from the OTP card, conversion in to gram per day was done. The cross-tab shows that out of 697 children whose data were available for therapeutic food consumption only 306 (43.9%) of them consumed therapeutic food according to their weight range. Among 373 children with admission weight of 5–6.9kg 56.3% of them were given inappropriate amount of RUTF which was between 105–130g/day irrespective of the standard (Table 4).

**Table 4:** Comparison of the study result with the standard of therapeutic food to be consumed.

Standard amount to be consumed per g/day	Proportion of children consumed RUTF per standard				total
	3–4.9 kg (n = __54___)	5–6.9 kg (n = ___373__)	7–9.9 kg (n = __250_)	10–14.9 kg (n = __36__)	
105–130g/day	45(6.4%)	210 (30.1%)	129 (18.5%)	28(4.0%)	412
200–260g/day		136 (19.5%)	16 (2.3%)	4(0.6%)	156
260–400g/day	4(0.6%)	22(3.2%)	97 (13.9%)	4(0.6%)	127
400–460g/day		1(0.1%)	1(0.1%)		2
Total					697

### Treatment outcomes and testing for statistical differences

Among 713 children with severe acute malnutrition enrolled in OTP, 569 children 79.8% recovered with a median time to recovery of 8.7 weeks (interquartile range: 5–14weeks), 11.2% defaulter, 3.8% were non-responders, 0.6% died, 2.1% transferred-out to other facility and 2.5% had an unknown outcome (Table 5). The median recovery time for children with SAM who were given RUTF inappropriate for their weight was 10.02 weeks, whereas for children with SAM who were given RUTF appropriate for their weight was 8.95 weeks.

**Table 5:** The comparison of the study results with international Sphere Standards, 2013–2016, Dire Dawa, Eastern Ethiopia.

Indicators	Results	The SPHERE project reference values [16]	
		Acceptable	Alarming
Recovery rate	79.8%	>75%	<50%
Death rate	0.6%	<10%	>15%
Defaulter rate	11.2%	<15%	>25%
Weight gain	6 g/kg/day	≥ 8 g/kg/day	< 8 g/kg/day
Length of stay	10.03 weeks	< 4 weeks < 8 weeks^۞^	> 6 weeks

^۞^*Unlike the sphere standard which set to be 4–6 weeks*, *the Ethiopian national protocol for management of SAM indicated for children to stay under OTP for a maximum of eight weeks*.

Recovery rate among children without comorbidity was (51.8%) and among children with certain medical problems were (48.2%). The average rate of weight gain among children without comorbidity was (6.11 gm/kg/day (95% CI = 5.42, 6.81)) and among children with certain medical problems were (5.89 gm/kg/ day (95% CI = 5.25, 6.54)). There was a significant difference in the recovery times between cohorts of children without comorbidity and children with comorbidity for a weight gain rate of ≥ 8g/kg/day (P-value<0.001).

**Fig 2:**
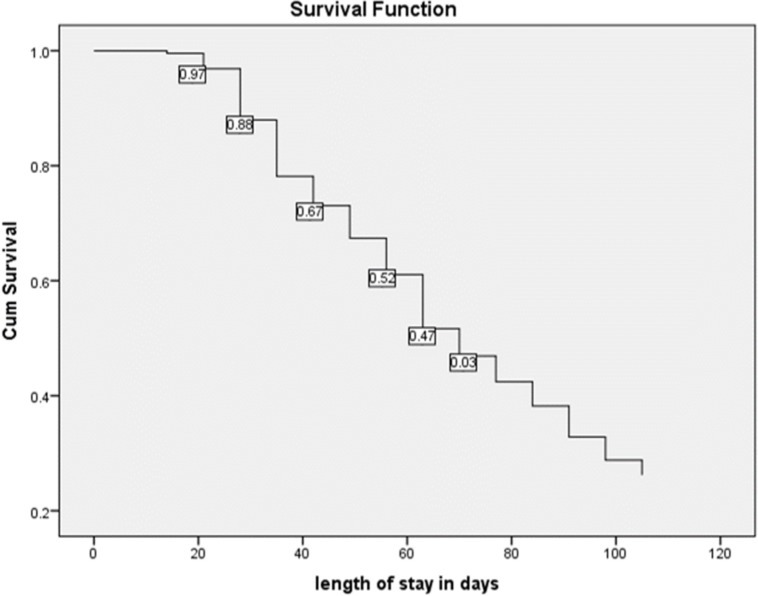
Overall Kaplan Meier recovery estimate of children treated under OTP in Dire Dawa, Ethiopia; January 2013 to December 2016.

The cumulative proportions of children surviving recovery at the end of the seventh week of treatment were 61% (Fig 2). The defaulter rate was 11.2% and the average defaulting time was 1.95 weeks (eg. most of the children was defaulting from the treatment within the first 7 days). The mean length of stay under OTP was 10.03 weeks (95% CI = 9.51, 10.55). However, the Ethiopian protocol for management of SAM limits the maximum length of stay in the treatment to be 8 weeks. The mean recovery time for children treated in rural health centers was 9.13 days (95% CI 8.4–9.86) and urban health centers was 10.73 days (95%CI: 10–11.46) (P-value = 0.002). The overall incidence rate of recovery with 95% CI was 17.23 (16.16, 18.29) per 100-person weeks observed. The incidence rates of recovery were 17.04 (15.44,18.64) for children treated in Rural health centers and 17.37 (15.94,18.80) for children treated in Urban health centers.

**Fig 3:**
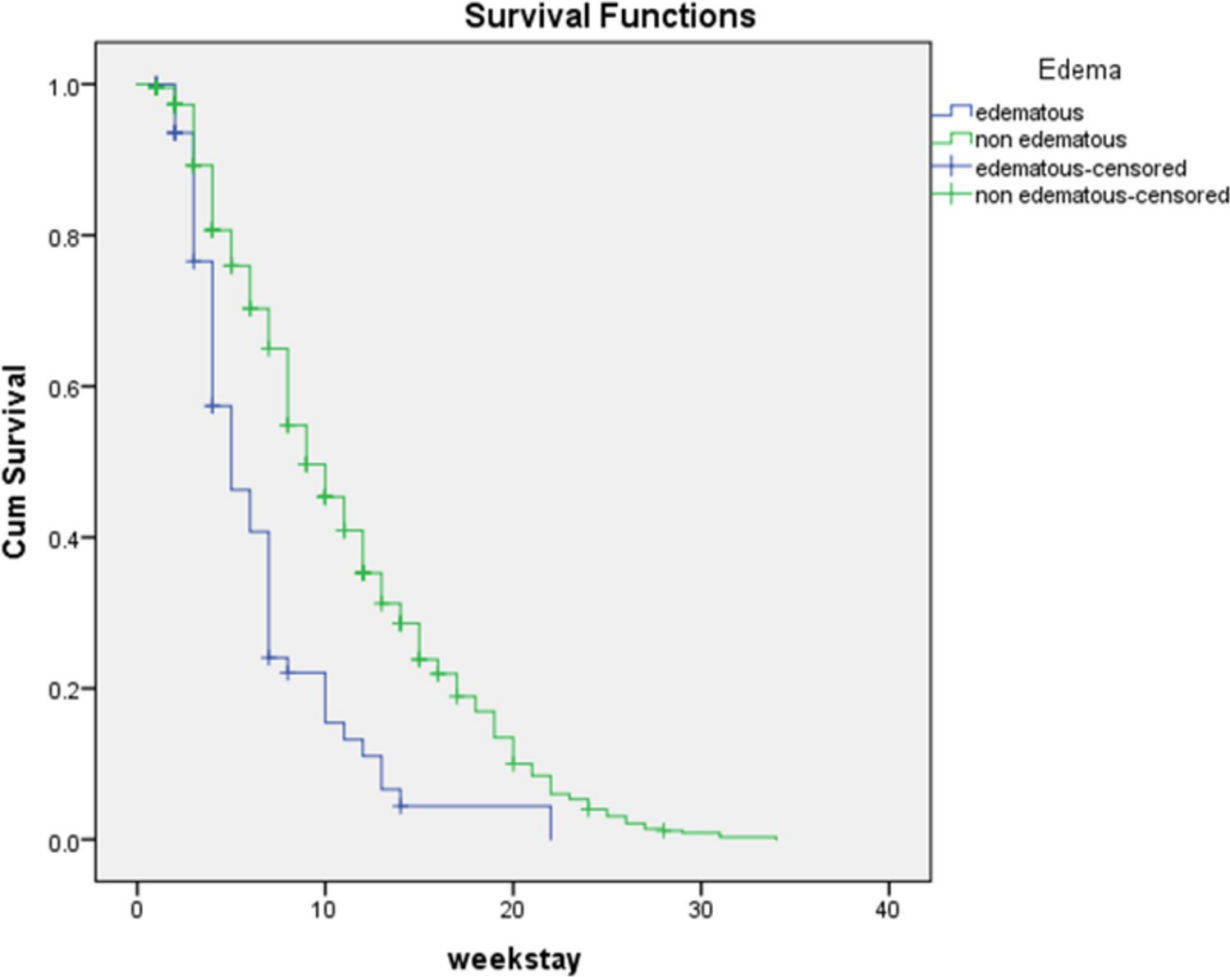
Comparative Kaplan Meir survival function for SAM Children with edema and without edema, from 2013 to 2016 in Dire Dawa, Ethiopia (Log Rank chi-square = 35.760, p-value < 0.001).

Log rank test were used to estimate survival curve (time to recovery) and to test for any observed difference in survival (recovery rate) curves between each grouped factor. There were significantly different in time to recovery among children who were treated in rural health centers and urban health centers (Log rank test, X2 = 9.478, P<0.05). Children with marasmus were 56% less likely to achieve nutritional recovery compared to children with edema (CHR = 0.44, 95% CI: (0.33–0.59). Further, children with marasmus (wasted) also took significantly longer time to recover than children with edema (Log rank test, X2 = 35.760, df = 1, P<0.001) (Fig 3).

### Predictors of time to recovery from SAM

In the bivariate analysis of selected baseline and management characteristics, children with marasmus (wasted) had 56% less likely to achieve nutritional recovery compared to children with edema (CHR = 0.44, 95% CI: (0.33–0.59)). Children who were being breastfed had 1.46 times more likely to recover faster than the reference (CHR = 1.46, 95% CI: (1.19–1.78), and those who had an admission weight of ≥7kg were more likely to recover faster (CHR = 1.30, 95% CI: (1.09–1.54). Moreover, a weight gain of ≥8g/kg/day (CHR = 5.69, 95% CI: (4.49–7.23)), children who took measles vaccine (CHR = 1.36, 95% CI: (1.15–1.62)), children who took Amoxicillin had 37% (CHR = 1.37, 95% CI: (1.13–1.66)) and the hazard of recovering faster from SAM is 80% higher for children who were dewormed (CHR = 1.80, 95% CI: (1.38–2.36)). In bivariate analysis, children who had cough (CHR = 0.81, 95% CI: (068–0.97)) (P < 0.05) and fever and on admission were less likely to achieve fast recovery from SAM (CHR = 0.72, 95% CI: (0.55–0.96)) (Table 6).

**Table 6:** Bivariate Cox-regression for prediction of time to recovery from SAM under OTP in Dire Dawa, Ethiopia; from January, 2013 to December, 2016.

Variables		Cured (%)	Censored (%)	CHR and 95% CI	P value
**Child’s age**	< = 24 months	442(62.0%)	116(16.3%)	1	0.029*
	>24 months	127(17.8%)	28 (3.9%)	1.25 (1.02–1.51)	
**Residence**	Rural	249(34.9%)	61(8.6%)	0.78 (0.66–0.92)	0.004*
	Urban	320(44.9%)	83 (11.6%)	1	
**Sex**	Male	284(39.8%)	70(9.8%)	1	0.841
	female	285(40.0%)	74(10.4%)	0.98 (0.83–1.16)	
**Breast feeding**	yes	307 (43.1%)	84 (11.8%)	1.46 (1.19–1.78)	< 0.001*
	No	134 (36.7%)	31(8.4%)	1	
**Edema**	Yes	53(7.4%)	12(1.7%)	1	< 0.001*
	No	516(72.4%)	132(18.5%)	0.44 (0.33–0.59)	
**Admission weight**	<7kg	342(48.0%)	85(11.9%)	1	0.002*
	> = 7kg	227(31.8%)	59(8.3%)	1.30 (1.09–1.54)	
**Cough**	Present	186(26.1%)	34(4.8%)	0.81 (068–0.97)	0.019*
	Absent	383(53.7%)	110(15.4%)	1	
**Diarrhea**	present	184(25.8%)	38(5.3%)	0.98 (0.83–1.18)	0.870
	absent	385(54.0%)	106(14.9%)	1	
**Folic acid**	Yes	40(8.8%)	1(0.2%)	1.04 (0.75–1.45)	0.807
	No	314(71.7%)	88(19.3%)	1	
**Vitamin A**	Yes	288(37.6%)	37(4.6%)	1.19 (1.01–1.41)	0.038*
	No	281(42.2%)	107(15.6%)	1	
**Amoxicillin/Antibiotics**	Yes	424(59.5%)	69(9.7%)	1.37 (1.13–1.66)	0.001*
	No	145(20.3%)	75(10.5%)	1	
**Measles**	Yes	269(37.7%)	33(4.6%)	1.37 (1.15–1.62)	<0.001*
	No	259(36.3%)	93(13.0%)	1	
	Not Indicated	41(5.8%)	18(2.5%)	1.02 (0.74–1.42)	0.888
**Albendazole/Mebendazole**	yes	134(18.8%)	14(2.0%)	1.80 (1.38–2.36)	< 0.001*
	No	344(48.2%)	98(13.7%)	1	
	Not indicated	91(12.8%)	32(4.5%)	0.99 (0.79–1.25)	0.937
**RUTF consumption** **(g/day)**	Appropriate for weight	269(38.6%)	37(5.3%)	1	0.045*
	not appropriate for weight	288(41.3%)	103(14.8%)	0.84(0.71–0.99)	
**Weight gain (g/kg/day)**	<8g/kg/day	419(58.8%)		1	< 0.001*
	≥8g/kg/day	150(21.0%)		5.704 (4.56–7.24)	

* show statistically significant association between predictors and recovery (P < 0.05).

The covariates having P<0.05 in the bi-variate Cox regression model were entered in to the multivariate Cox Regression model. For clinical significance and control of confounding, age, sex and medication were included in the multivariate Cox regression. Controlling for the effects of admission weight, age, sex and medication; children with marasmus were shown to be 49% less likely to achieve nutritional recovery compared to children with edema (AHR = 0.51, 95% CI: (0.37–0.71). Children with an admission weight of ≥7kg were 1.7 times more likely to recover faster compared to those who had <7kg (AHR = 1.73, 95% CI: (1.49–2.14)), children who were dewormed were 1.4 times (AHR = 1.44, 95% CI: (1.01–2.07)) more likely to achieve nutritional recovery compared to those who did not take them. Children who did not consume Plumpy Nut (g/day) according to their weight were 22% less likely to achieve nutritional recovery compared children who consumed the appropriate amount of Plumpy Nut (AHR = 0.78, 95% CI: (0.65–0.94)). Children who gained ≥8g/kg/day were 5.8 times higher probability to recover faster compared to those who did not (AHR = 5.81, 95% CI: (4.54–7.43)), (P value < 0.001) (Table 7).

**Table 7:** Multivariate Cox-regression for prediction of time to recovery from SAM under OTP in Dire Dawa, Ethiopia; from January, 2013 to December, 2016.

Variables		Cured (%)	Censored (%)	CHR and 95% CI	AHR and 95% CI	P value
**Edema**	yes	53(7.4%)	12(1.7%)	1	1	<0.001*
	No	516(72.4%)	132(18.5%)	0.52(0.37–0.71)	0.51(0.37–0.71)	
**Admin weight**	<7kg	342(48.0%)	85(11.9%)	1	1	<0.001*
	> = 7kg	227(31.8%)	59(8.3%)	1.72(1.39–2.13)	1.73(1.41–2.14)	
**Measles**	Yes	269(37.7%)	33(4.6%)	2.09(1.36–3.20)	1.49(0.98–2.27)	0.059
	No	259(36.3%)	93(13.0%)	1	1	
	N/A	41(5.8%)	18(2.5%)	1.41(0.79–2.49)	0.71(0.41–1.27)	0.256
**Albendazole/Mebendazole**	Yes	134(18.8%)	14(2.0%)	1.44 (1.01–2.06)	1.44 (1.01–2.07)	0.047*
	No	344(48.2%)	98(13.7%)	1	1	
	N/A^+^	91(12.8%)	32(4.5%)	0.89 (0.66–1.21)	0.89(0.65–1.21)	0.463
**RUTF consumption (g/day)**	Appropriate for weight	269(38.6%)	37(5.3%)	1	1	
	not appropriate for weight	288(41.3%)	103(14.8%)	0.79(0.66–0.95)	0.78(0.65–0.94)	0.012*
**Weight gain (g/kg/day)**	<8g/kg/day	419(58.8%)		1	1	<0.001*
	≥8g/kg/day	150(21.0%)		5.76 (4.51–7.38)	5.81(4.54–7.43)	

*Statistically significant predictor of recovery (P < 0.05).

N/A: Not applicable and N/A^+^ not applicable i.e. children less than one year ages are not eligible to take de-worming tabs).

All the predictors in the table were adjusted for one another to control for confounding effect.

## Discussion

This study showed that the overall recovery rate was 79.8% (95% CI: 74.4% - 85.6%). 0.6% of participants died, 3.8% were categorized as non-responders, 2.1% transferred out, 2.5% were unknown and 11.2% were defaulters from OTP. The median recovery time was 8.7 weeks with an average rate of weight gain 6 g/kg/day. The major contributing factors related to the recovery time were admission weight, type of SAM, taking deworming, weight gain and consumption of Plumpy Nut.

The recovery rates were within the acceptable threshold for the Sphere International Standards which recommends >75% of recovery from SAM [16]. The recovery rate is almost similar with the study done in Shebidebo which showed a recovery rate of 78.7% [10] and in Enderta district of Tigray where the recovery rate was 76.8%. However, it was higher than the study done in Wolaita zone 37.8% [7], 67.7% in Kamba [11], 68.8% in Sidama [13], 61.78% in Tigray [12], in Kitui County Kenya which reported 73.3% [17] and in India 53.4% [18]. These discrepancies might be due to differences in adherence to management of SAM protocol in different regions, unequal accountability between children’s parents for their household tasks, way of preparing and feeding foods, sharing of the RUTF with the healthy children found at home and weak educational infrastructure might be the possible explanation for poor recovery rate. Reports show that sharing of Plumpy Nut with healthy children at home, feeding only Plumpy Nut, traditional way of preparing food causes extensive hydrolysis of the protein and carbohydrate. Caretakers (women) engaged in household tasks and striving for income reduces optimal care for the children and illiteracy led to limited awareness about the importance of health, hygiene and nutrition.

The median recovery time was 8.7 weeks (61 days) and the mean length of stay was 10.03 weeks (72days). It was outside of the acceptable international sphere standard which is < 4 weeks [16] as well as the standard of the Ethiopian protocol for management of SAM which allows children to stay under treatment up to 8 weeks [2]. The length of stay was also higher than other similar studies done in Kamba South Western Ethiopia and Sidamo Southern Ethiopian which was 7.14 weeks (50 days), in Shebidebo 42 days [10], in Tigray 6.24weeks (43.68 days) [12], in Ghana 8 days [19] and in India report showed that mean length of stay was 8.7 weeks [18]. The reason for this high estimated length of stay under OTP could be not following the management protocol for SAM children. This study shows 347 (61%) of cured children were allowed to stay for more than 8 weeks under OTP. According to the national Ethiopian protocol for SAM management under OTP [2], these children were supposed to be referred to hospitals or other health facility which have stabilization centers (SC) service under inpatient therapeutic feeding center when they failed to reach any of the discharge criteria [2]. Additionally, in this study most of the children 391 (56.1%) did not consume therapeutic food according to their weight range. Low provision of RUTF could cause delay recovery time in OTP [7].

The average rate of weight gain was 6 g/kg/day which is lower than the international sphere standard which is (≥8g/kg/day) [16]. It was also lower than findings from studies carried out in Ghana which shows 28.3g/kg/day [19] and Tigray region of Ethiopia 8.3g/kg/day [20]. However, it was higher than study done in Kitui County Kenya 5.1 g/kg/day [17], study done in India which showed 4.7 g/kg/day and in Shebidebo which was 3.85g/kg/day [10]. These discrepancies could have been due to the inappropriate management of SAM such as partial prescription of routine medication and inadequate provision of Plumpy Nut by service providers which results in low average weight gain and causes the children to stay longer than the recommended period of time in the program to recover from SAM. Additionally, the result of this study shows children were not given RUTF according to their body weight and most of the children were given 105 – 200g/kg/day. So RUTF should be given according to the standard set in SAM management protocol.

In this study, being dewormed had 1.44 times more likely to recover faster compared to those who did not take them, this is in line with the study done in Tigray [12]. This might be due to the fact that children with SAM are at increased risk of infection and treating children with deworming may lead to better absorption of nutrients and an improved appetite. This study also revealed that children who did not consume Plumpy Nut (g/day) according to the protocol were 22% less likely to achieve nutritional recovery compared to children who consumed Plumpy Nut according to the protocol. It is supported and in line with the fact that Plumpy Nut is suited for SAM children treated at OTP, which also indicated that taking Plumpy Nut increases the recovery rate from SAM.

Admission weight with time to recovery were significant in this study. Children with an admission weight of ≥ 7kg were 1.7 times more likely to recover faster than children with an admission weight of <7kg (AHR = 1.72). This study also shows among children with <7kg admission weight 48% of them were given inappropriate amount of RUTF which was between 105–130g/day and this lower provision of Plumpy Nut beyond the standard set in management protocol for SAM [2] could delay the recovery time. The study done in Shebidebo reported lack of significant association between recovery time and admission weight [10]. The finding of this study can be possibly explained by insufficient attention paid to children with a lower admission weight by health care providers and in this study 64.8% of children who had <7kg of admission weight were with medical problems, which makes them more susceptible for not recovering quickly. It was reported that severely malnourished younger children are more vulnerable to infections, because of depressed immunity leading to mal-absorption of nutrients and insufficient feeding practices.

This study shows that children with marasmus were 49% less likely to achieve nutritional recovery compared to children with edema. This finding was consistent with the study done in Shebidebo [10]. This is potentially owing to the fact that marasmic child experience severe muscle wasting and severe weight loss and develop slowly. As such, it may take a longer time to recover than children with edema. This study also showed that weight gain was significantly associated with median recovery time in which children who gained ≥8 g/kg/day were 5.8 times more likely to recover earlier compared to those who gained < 8 g/kg/day (AHR = 5.81). This is supported and in line with the recommendations of the Ethiopian protocol for the management of SAM [2] as well as the international sphere standard [16] which indicates weight gain to be more than 8 g/kg/day. These also shows that weight gain has a strong correlation with time to recovery.

Another reason for poor recovery rate and delayed recovery time from the interviewed data were having additional co-morbidities, availability of Plumpy Nuts, distance from their home to health center, lack of awareness on how to use Plumpy Nut, affect the recovery rate.

A 25-year old female nursing said,” *When we are out of stock for Plumpy Nut*, *they stop coming back*, *once they are gone home empty handed”*. As one 55-year old male nurse said, *“their mothers/caregivers terminates the treatment course due to the place where the health center found*, *which is far in distance for some of the community members*.*”*

The finding was similar with the qualitative study done in Wolayta, Ethiopia. The study found that inadequate provision of RUTF by the service providers leads to poor treatment outcome of SAM children treated under OTP [7] Similarly, a study done in Bangladesh found that availability of medicines in the health centers had an important influence on the attendance and according to health workers, the attendance drastically decreases with RUTF shortages [21].

Majority of the participants in the interview mentioned that, sharing of Plumpy Nut at home contribute to delayed recovery from SAM. As a 38-year old female nurse said, *“the mothers/caregivers are sharing the Plumpy Nut to other healthy child found at home and sailing of the Plumpy Nut given to their sick child like a source of income*.*”* The finding were similar with a qualitative study done in Wolayta which reports that RUTFs were available in shops and sharing of the RUTF with the healthy children found at home have the potential to delay the nutritional recovery of SAM children and this leads to chronic nutritional deprivation of the children [7].

This study was based on secondary data, so that some missing data and inconsistency in outpatient record cards were some of the problems. Also, some variables like distance (time of travel) from home to health facilities, malaria, hypothermia and height was not found in some incomplete cards and registration. There was also poor registration and documentation in the program.

## Conclusions

The recovery rate was within the acceptable ranges of International Sphere Standards. Experiencing a lower rate of weight gain and a higher average length of stay among enrolled SAM patients were identified as major problems for the program effectiveness. Diarrhea, cough and others were the most common medical complications affecting children with SAM. Admission weight, edematous type of SAM, being dewormed, weight gain more than 8 g/kg/day and consumption of Plumpy Nut were found to be significantly associated with time to recovery from SAM, and can be concluded as there are gaps in following the protocol for management of SAM.

## Recommendations

Moreover, there were gaps in adherence to SAM management protocol in treating children under OTP at the study area. As this study illustrated, insufficient attention to children with lower admission weights and low provision of Plumpy Nut affects recovery time from SAM. Therefore, health care providers should give a special focus to children who have lower admission weights in OTP. At the site of OTP, give deep nutritional counselling for caregivers that Plumpy Nut is not meant to be a substitute for food and should not be given for healthy children found at home. Provide nutrition education at community level through health extension workers to prevent child under-nutrition and miss use of Plumpy Nut. All concerned bodies should give emphasis on RUTF supply in the region as well as at national level to avoid partial provision of RUTF. For researchers further qualitative and prospective studies should be conducted that include more at community level to generate more and deep information to improve and strength the program.
